# Enhancing a peer supporter intervention for young mothers living with HIV in Malawi, Tanzania, Uganda, and Zambia: Adaptation and co-development of a psychosocial component

**DOI:** 10.1080/17441692.2022.2081711

**Published:** 2022-05-29

**Authors:** Christina Laurenzi, Agnes Ronan, Lynn Phillips, Sharifah Nalugo, Eugene Mupakile, Don Operario, Elona Toska

**Affiliations:** a Institute for Life Course Health Research, Department of Global Health, Stellenbosch University, Tygerberg, South Africa; b Paediatric Adolescent Treatment Africa, Cape Town, South Africa; c Department of Behavior and Social Sciences, School of Public Health, Brown University, Providence, RI, USA; d Centre for Social Science Research, University of Cape Town, Cape Town, South Africa; e Department of Sociology, University of Cape Town, Cape Town, South Africa; f Department of Social Policy and Intervention, University of Oxford, Oxford, UK

**Keywords:** Adolescent motherhood, adolescents living with HIV, peer support, co-development, psychosocial support

## Abstract

Young mothers living with HIV (YMHIV) experience heightened risks to their mental health, as their transition to adulthood is marked by social stigma, health and socioeconomic challenges. Targeted psychosocial interventions may improve the mental health of YMHIV; however, no evidence-based interventions have been developed for this group. Peer support models, more common for youth living with HIV, show promise as a design to reach YMHIV in a non-stigmatising way. This manuscript describes the process of adapting and co-developing an evidence-based psychosocial component (Boost) of a larger intervention called *Ask-Boost-Connect-Discuss*. Peer supporters in Malawi, Tanzania, Uganda, and Zambia used ABCD to guide group sessions with YMHIV. The research team partnered with an implementing partner, Paediatric-Adolescent Treatment Africa, to undertake this work in three phases: 1) formative research, 2) content adaptation and development, and 3) consultation, refinement, and modification. YMHIV (n = 4), peer supporters (n = 21), and technical advisors (n = 4) were engaged as co-developers, shaping the resulting Boost intervention component at each phase. Peer support models may effectively reach young mothers, and consultation, co-creation, and integration with existing programming can offer rich insights to inform these models. We discuss the implications and promise of this approach.

## Introduction

Young women living with HIV face a complex set of challenges at the intersection of HIV, early motherhood, and mental health. In sub-Saharan Africa, 83% of new HIV infections are among 15-to 24-year-old adolescent girls and young women (UNAIDS, 2021). While grappling with their HIV-positive status, many of these young women are also navigating sexual and romantic relationships, unintended pregnancies and early motherhood (Busza et al., 2013; Toska et al., 2020). Based on nationally representative data from several sub-Saharan African countries, one in five young women in the region have had a child before the age of 18 (Williamson, 2013). Notably, young mothers living with HIV have been found to face poorer health outcomes than older mothers, including in areas of healthcare engagement, prevention of mother-to-child transmission (PMTCT), and postpartum HIV treatment; they also have higher rates of HIV transmission to their children (Horwood et al., 2013; Nuwagaba-Biribonwoha et al., 2018; Ronen et al., 2017).

Young mothers living with HIV also experience heightened risks to their mental health, including increased stigma, due to their HIV status and early pregnancy and motherhood (Roberts et al., 2021). Qualitative research from sub-Saharan Africa indicates high levels of shame and blame associated with unintended pregnancy and early motherhood, particularly among unmarried mothers, leading to social isolation and reduced contact with peers (Fielden et al., 2011; Hall et al., 2018; Josephine, 2019; Kumar et al., 2018; Levandowski et al., 2012; Logie et al., 2019; Moodley, 2021). Concurrently, young mothers are managing stigma and stress related to HIV: many of them learn their HIV status at the same time they learn about their pregnancies (Nuwagaba-Biribonwoha et al., 2018). As a result, these stigma experiences are often complex and layered. Quantitative analyses indicate that poor mental health among young people living with HIV is strongly associated with reduced retention in HIV care (Pantelic et al., 2020). Context, including structural, cultural, and geographical factors, can also play a central role in shaping mental health needs and available psychosocial support, which affect health and HIV outcomes (Roberts et al., 2021).

Evidence-based interventions that respond to the distinct needs of young mothers living with HIV and navigating early motherhood are also scarce. Existing psychosocial support interventions targeting a broader cross-section of mothers living with HIV in Botswana, Malawi, and Zimbabwe have found improved HIV outcomes and healthcare engagement among older mothers (Ronan et al., 2020), with more limited uptake and effectiveness among younger mothers (Kossow et al., 2012; Orne-Gliemann et al., 2017). However, to our knowledge, no interventions have specifically targeted psychosocial wellbeing and improved mental health outcomes among young mothers living with HIV. Acceptable, feasible and effective interventions that address the psychosocial needs of young mothers living with HIV are urgently needed (Laurenzi et al., 2020a, 2020b).

While no targeted interventions for improved mental health among young mothers living with HIV have been tested, increasingly, peer-facilitated models are seen as a promising delivery strategy for task-shifting HIV prevention and care – including psychosocial support – in overburdened and resource-constrained healthcare systems (Casale et al., 2019; Mark et al., 2019). Peer support is a critical strategy to improve adolescent- and youth-friendly health services for adolescents and young people living with HIV, helping them to access, engage with and be retained in treatment. For young people in particular, peers can be a unique and powerful source of empathic support. Peer supporters can positively influence behaviour, as they function as credible and affirming role models, sharing similar experiences and/or backgrounds and helping young people living with HIV to feel connected. These kinds of engagements have also been found to increase awareness of positive coping strategies among young people receiving peer support (Foster et al., 2016; Willis et al., 2019).

There is a small but growing evidence base on the acceptability and effects of peer-led psychosocial interventions to promote improved physical and mental health outcomes among young people living with HIV (Ferrand et al., 2017; Simms et al., 2022; Willis et al., 2019). Peer supporters, themselves living with HIV, may be particularly well-positioned to connect with young mothers and address the social and internalised stigma they face. However, as with other lay health workers, they need adequate training and support to do so (Laurenzi et al., 2021b). As HIV infections and early pregnancy among adolescent girls and young women persist despite ongoing prevention efforts, it is critical to identify effective pathways for intervening to support the mental health of young mothers living with HIV. Importantly, interventions that are locally adapted and age-appropriate, yet based in the most up-to-date scientific evidence, need to be developed and tested. Through engaging with a peer support model implemented by Paediatric-Adolescent Treatment Africa (PATA, see Box 1), we set out to respond to this urgent gap in evidence and practice.

### Enhancing the peer support model with Ask-Boost-Connect-Discuss

In 2017, PATA developed and rolled out a peer supporter training manual, which included a component on providing psychosocial support to adolescents and young people living with HIV (Paediatric Adolescent Treatment Africa (PATA), 2017). During the rollout of this manual, peer supporters in several clinics provided feedback on additional specific skills they felt they needed to be able to provide support to their peers (PATA, 2019). In particular, young mothers living with HIV were identified as particularly at risk of poor physical and mental health, experiencing overlapping psychosocial burdens linked to motherhood and HIV.

Using PATA’s existing peer support model, a set of additional components was devised to effectively upskill peers to support young mothers with their psychosocial needs and identify appropriate services for referral. Drawing on task-shifting approaches used with other lay health worker interventions, these components were combined as a four-part, multi-layered intervention called **Ask-Boost-Connect-Discuss (ABCD),** comprising:

- **Ask:** a screening phase at intake, at which time peer supporters could assess the eligibility and needs of the young mother;

- **Boost:** modular, evidence-based content, envisioned to be light-touch and easily communicable for and by non-specialists;

- **Connect:** protocols for peer supporters to connect young participating mothers to onwards services and referrals;

- **Discuss:** supervisory functions for peer supporters, including performance review during regular meetings between peer supporters and supervisors, and ongoing opportunities for communication on an as-needed basis.

This manuscript describes the adaptation and co-development of the **Boost** component in particular; the modular content envisioned for **Boost** was designed to guide peer supporters to deliver this psychosocial component to young mothers living with HIV at health facilities. We draw on end-user consultations and implementation data to describe the process of adapting and co-developing the **Boost** component of the ABCD intervention in preparation for its implementation across four sub-Saharan African countries.

## Methods and findings

Between 2018-2019, we adapted and co-developed a psychosocial component, **Boost**, of a larger mental health-focused intervention, in partnership with young mothers living with HIV (n = 4), peer supporters living with HIV (n = 21), and technical advisors (n = 4) in Malawi, Tanzania, Uganda and Zambia. We coordinated this process closely with PATA’s implementation and advisory teams, and devised steps around existing methods for intervention adaptation, explained in more detail below (Rahman, 2007).

### Participants and study setting

Country selection for developing and testing this intervention was purposeful, linking with PATA’s existing networks of peer supporters. The implementation team included countries from Eastern and Southern Africa to account for service-provision context and HIV epidemic variations and understanding of adaptation in different cultural contexts. Health facilities in two East African countries (Uganda, Tanzania) and two Southern African countries (Malawi, Zambia) were chosen specifically due to factors including their existing capacity to support peer supporters; their physical location and proximity to other participating sites; and the existing relationships and ability of technical advisors to travel to each site and support ongoing programming.

In addition to the project team, including PATA staff and researchers from two partner universities, a number of stakeholders took part across the continuum of co-development of the tool. These stakeholders included the following participants:

- **Young mothers living with HIV:** A small number of young mothers living with HIV were engaged in early-stage consultations (n = 4). To be eligible, young mothers had to be between the ages of 18-24, living with HIV, currently pregnant or having had delivered a child within the last 3 years, and receiving care at a PATA-supported facility.

- **Peer supporters:** Peer supporters were adolescent and young women living with HIV between the ages of 18–24 who facilitated peer support groups prior to the start of the project, under existing PATA programming. For this specific project, only female peer supporters were enlisted to engage in the development and delivery of the intervention, and only peer supporters who were linked to pre-selected clinics were invited to participate. Peer supporters did not have to be mothers themselves. Peer supporters were engaged in preliminary consultations through written feedback shared via email (n = 2) and a larger group helped test the tool, providing feedback on both context and structure of the intervention package (n = 21).

- **Technical advisors:** Technical advisors (n = 5) were adult professionals employed by PATA; there was one technical advisor in each of the focus countries, plus one additional advisor from Kenya involved in the consultation phase. These individuals had varied expertise (including psychiatry, nursing, social work, and programme management). These individuals were responsible for providing mentorship to peer supporters and acting as a communication channel between the peer supporters and PATA. Technical advisors were engaged along all phases to provide contextually specific information, review content, and offer suggestions for refinement. Data stemming from these individuals included consultation notes, debriefing notes, and email communications.

### Ethics

Ethical approval for analysis of the programmatic data collected with the above stakeholders during the three phases described below was granted by the University of Cape Town (Sociology REC2020/10/11). Voluntary informed consent was obtained from all individual participants, and authors confirmed with all programmatic staff that they consented to programmatic data being analyses as part of this research.

### Data analyses

The first and last author reviewed all data sources, distilling emerging themes and consulting with all other authors who comprised the implementation team, technical advisors and peer supporters. Table 1 shows an overview of data sources across each of the three phases.

### Process and procedures

**Figure 1.**
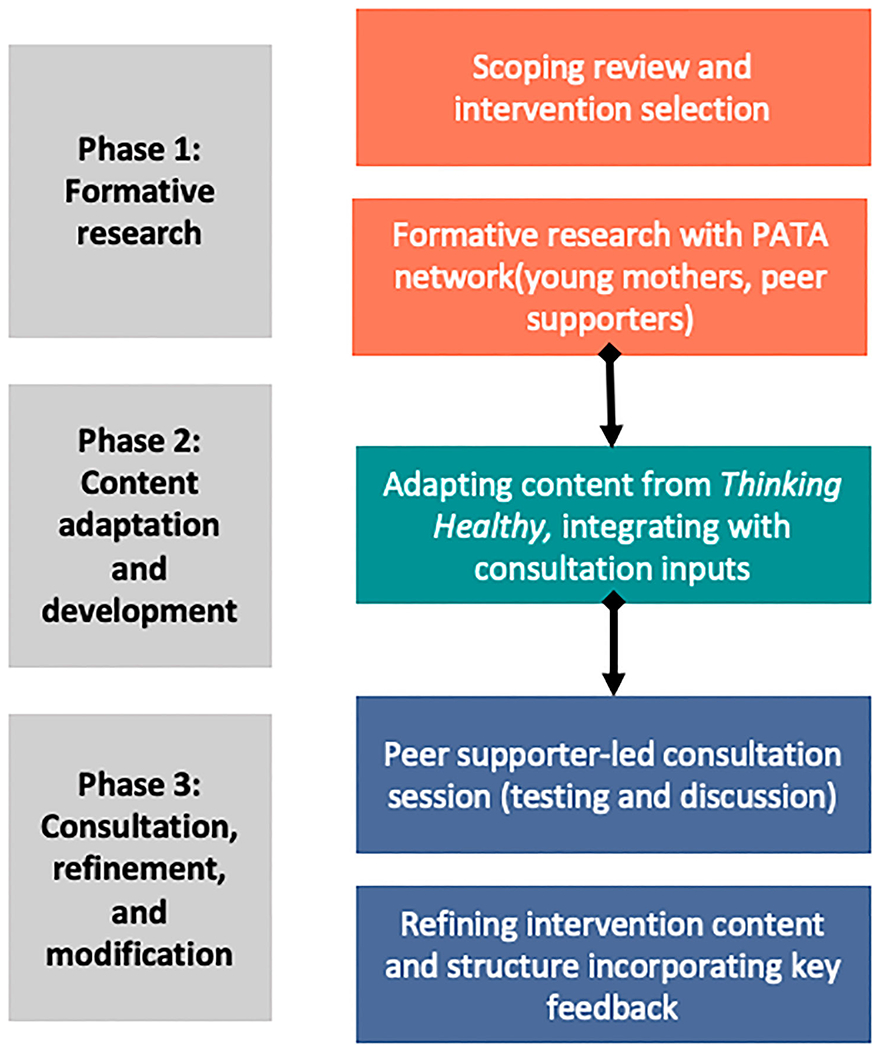
Flow of intervention adaptation and co-development.

The co-development process consisted of three overall phases, with methods for each described in detail below and summarised in Figure 1. Our methods and findings are combined in the sections that follow, to facilitate a more nuanced report of how this process was conducted.

### Phase 1 methods: Formative research (August-September 2018)

#### Scoping review.

To become familiar with the existing evidence, the research team conducted a scoping review including reviewing peer-reviewed literature, programmatic data, and expert consultations to identify existing modes of providing peer-delivered psychosocial support. Specifically, we sought out intervention approaches that 1) were implemented by a layperson, 2) found to be effective for improving mental health among at-risk mothers, and 3) had produced evidence from large-scale implementation efforts in low- and middle-income countries (LMICs). The aim of this scoping review was to identify interventions that might be suitable for adaptation and integration into the ABCD model, to form the basis of the **Boost** component.

#### Contextualisation with the PATA network.

In addition to exploring existing intervention approaches, the research team sought to incorporate input from the broader PATA network about acceptable modes of delivering psychosocial support to contextualise findings. The lead researcher and first author (CL), who had prior expertise in adolescent and youth mental health interventions, led this process, reviewing potential intervention modules and mapping areas for further inquiry and potential content adaptation.

The process of contextualisation was embedded within project activities, enabling a site visit to one country only. We developed a set of questions for young mothers living with HIV in Uganda and for Ugandan peer supporters in the PATA network; Uganda was chosen as a country with a strong peer supporter team. Questions asked about topics including understandings of mental health and ways to confront psychosocial challenges (including help-seeking); common fears, concerns, anxieties among young pregnant women; and common mechanisms or thoughts to confront these fears. Remaining questions were context-specific open-ended questions about pregnancy and HIV knowledge; common experiences for young mothers (e.g. school enrolment, marriage, and financial support); traditional beliefs or practices surrounding childbearing, early infancy, mental health and maternal mental health; and specific services available for mental health care and treatment.

These questions were designed to be asked in a focus group setting, but were also made to be flexible to allow for informal individual interviews and written responses. Informal in-person interviews were conducted by a PATA programme officer during a site visit, in English where possible and with a translator when required. To increase representation across countries, questions were also circulated via email to technical advisors for distribution to peer supporters and select young mothers living with HIV across all four countries.

The first author listened to each audio recording and took summary notes of key lessons and takeaways reported in this manuscript. Preceding interviews, young mothers gave verbal consent; audio files were de-identified later during review and note-taking.

### Phase 1 findings

#### Scoping review.

Informed by a 2013 systematic review (Rahman et al., 2013) and the scoping review findings, we ultimately identified and selected the WHO-endorsed *Thinking Healthy* intervention to inform the development of **Boost**. *Thinking Healthy* was attractive for several reasons and fit the criteria established for intervention selection. The intervention is based on cognitive behavioural therapy techniques, and is delivered by lay health workers to reduce perinatal depression and improve child outcomes (Atif et al., 2017). Implementing lay health workers are instructed to recruit and follow pregnant women at various stages over the course of their pregnancy and into the child’s early infancy, across 16 themed sessions that focus on the mother’s personal health, her relationship with the baby, and her relationship with people around her (World Health Organization, 2015). Through this structure, lay workers help their clients to identify unhelpful or unhealthy thinking patterns and replace them with helpful or healthy thinking. *Thinking Healthy* was initially co-designed with numerous stakeholders globally and in South Asia, including end-users, to ensure relevance and effectiveness in targeting perinatal mental health; it relied on well-tested, evidence-based approaches to reach mothers in engaging ways (Rahman, 2007; Rahman et al., 2008). It has been adapted to diverse cultural and social contexts, and rolled out in low- and middle-income settings (Atif et al., 2017) including Pakistan, India, and Vietnam (Fisher et al., 2014; Fuhr et al., 2019; Maselko et al., 2015).

While the original *Thinking Healthy* intervention was developed for a more general maternal audience, its principles, structure, and content provided an important foundation for conceptualising how to deliver a peer-led intervention for young mothers. *Thinking Healthy* has also been well-integrated into existing health system structures (e.g. home visiting by trained village health workers) in settings where it has been successfully implemented. As PATA, too, was embedded in clinics across the four focus countries, facility- and community-level integration was an important consideration to connect with young mothers easily.

In subsequent consultations with PATA’s implementation and advisory teams, we confirmed that the intervention structure and content of *Thinking Healthy* would be a good fit for adapting enhanced content for **Boost**, to layer on top of PATA’s peer support programme.

#### Contextualisation with the PATA network.

Preliminary information was requested across all four countries, with a site visit in Uganda for more in-depth data collection. Findings from informal interviews (n = 3) and focus group discussions with young mothers (n = 1), as well as completed forms with written feedback from peer supporters (n = 2), helped to contextualise initial content scoping and adaptation efforts—despite the fact that only participants in Uganda contributed to this phase.

#### Peer supporter perspectives.

Poor mental health was sometimes seen as a person being ‘a failure in life’ (Uganda, peer supporter 1) or accompanied by the widespread notion that those people with mental health problems ‘have a problem in their brain and deserve no special attention’ (Uganda, peer supporter 2). Similarly, pregnancy before the age of 18 was strongly discouraged, or considered ‘an offence’ by health workers and family and often led to permanent school dropout (Uganda, peer supporter 1, 2). Suggested positive re-framings included working hard to achieve life goals (Uganda, peer supporter 1) and identifying a few promising pathways towards skills-building and livelihood support, such as tailoring or becoming a seamstress (Uganda, peer supporter 2).

#### Young mothers’ perspectives.

Engagements with young mothers revealed an additional set of considerations surrounding early motherhood and HIV. These issues included stigma surrounding unintended pregnancy in particular, especially from families in cases where the pregnancy brought shame on the family. One interviewee noted that those people she could have relied upon in earlier days would now refuse to help her because she had committed a wrong by becoming pregnant; she further explained that no one had ever discussed with her mental health or well-being linked to her pregnancy (Uganda, interview 1 notes). Young mothers also spoke emotionally about the persistence of socioeconomic challenges stemming from a lack of structured support; unable to rely on their families, young women sometimes also experienced abandonment by the father of their child. In a few instances, they became reliant on a new boyfriend to support them with their own and their child’s needs, leading to rapid repeat pregnancies that might repeat the cycle.

Patterns of negative thinking were also identified around the pressure placed on mothers to safeguard their infants; that if something happened to the child, the mother had not done enough to prevent this, and that God also had a role to play in the baby’s health (Uganda, interview 1 notes). Similarly, this interviewee said she drew strength from having her baby to talk to and play with, despite anxieties around financial stability.

### Phase 2 methods: Content adaptation and development (September-November 2018)

The formative research described in Phase 1 informed the process of content adaptation and development. Incorporating data from the early-stage scoping review and contextualisation efforts, the first author reviewed modules from the selected intervention, *Thinking Healthy* drafted adaptations to module content, and devised a structure for the **Boost** intervention modules that would mirror the selected intervention modules and delivery. Early drafts of this content and structure were reviewed and refined by PATA collaborators and members of the implementation team, ahead of broader consultation at the PATA Youth Summit in November 2018 (see more information in Phase 3). Key implementation-related considerations included language, delivery mode, framing of content, and existing infrastructure within which the intervention would be embedded in all four countries. Routine meetings provided opportunities for content to be adapted and refined in consultation with the broader team.

### Phase 2 findings

Incorporating data from formative research with technical advisors and young mothers living with HIV, the research team focused on adapting aspects of the *Thinking Healthy* intervention linked to content and visuals, session dosage and delivery, and format for the **Boost** component.

#### Content and visuals.

Content areas identified through the responses from technical advisors and young mothers were mapped onto existing *Thinking Healthy* modules and topics. A revised content workflow and structure was designed to inform the intervention. Content was selected that reflected both the original content in *Thinking Healthy*, and also the needs and specific challenges facing young mothers shared in the formative research. Module themes included: identifying factors within and outside of one’s control; reducing self-blame around motherhood; support-seeking practices; self-care during the transition to motherhood; child developmental milestones; managing stigma and judgement from others; practicing healthy behaviours; and future orientation while living with HIV. Prompts were introduced to connect each part of the intervention, and different colour text referred to prompts for the peer supporter to read aloud versus information for them to read to themselves as they went through the programme. Content for **Boost** sessions was translated into Kiswahili (for Tanzania) and was anticipated to be delivered in English in Malawi, Uganda, and Zambia.

Images in the *Thinking Healthy* manual, which was first developed for use in Pakistan, were largely geared towards Middle Eastern and South Asian mothers. A set of the original images were identified for adaptation so that they would resemble young African mothers, employing adolescent-friendly and engaging graphics in which young mothers and peer supporters could see themselves mirrored. A graphic designer was employed to produce these images for the ABCD intervention, to be inserted alongside **Boost** content.

#### Session dosage and delivery.

Following conversations about availability, need, and how to engage adolescents and young women most effectively, the research team decided to streamline the number of sessions, rather than delineating sessions by stage of pregnancy as *Thinking Healthy* had done. One reason for this was to make the intervention more inclusive for young mothers across multiple ages and stages of pregnancy and infancy, based on input from peer supporters who voiced a desire to be able to meet the needs of mothers when they came for postnatal visits, infant immunisations, or HIV-related appointments. At this stage, eight sessions were drafted: it was intended that individual participants would be recruited and engage with each session sequentially in one-on-one sessions with the peer supporter. Content for the first two sessions would also be tailored depending on whether the participant was 1) pregnant or 2) had recently delivered a child.

The structure of the original intervention, adopting a cognitive-behavioural approach to interrupting these unhealthy thought cycles, was retained. Furthermore, all participating young women would have the opportunity to select 1–3 unhealthy thoughts per session that they related to, and walk through a thoughts-feelings-behaviours scenario where they imagined how this thought affected emotions and behaviours, and where they discussed possible ways to change to healthy thinking. This approach was selected to give both structure and flexibility to individual participants as they worked through the session with their peer supporter. Hard-copy supplementary materials for participants (participant packs) were also drafted and printed, allowing participants to track their moods and record their activities between sessions.

### Phase 3 methods: Consultation, refinement and modification (November 2018-January 2019)

An extensive in-person consultation session for the principles and content of the intervention took place during PATA’s 2018 Youth Summit, an annual event for their network of young people, health professionals, and advisors, in Dar es Salaam, Tanzania. This consultation included two meetings that included, collectively, peer supporters (n = 21) from the four focal countries and one additional country (Kenya), and a group of technical advisors (n = 5), all of whom provided feedback on the principles and content of the intervention.

First, peer supporters and technical advisors were given a brief presentation that included a psychoeducational component and shared the proposed intervention’s structure and format. Second, peer supporters were given the opportunity to test the intervention content. Third, an open discussion took place, during which peer supporters shared their perceptions of the delivery structure, and specific programme content. Specific feedback was sought about the suitability of the content and session topics, the manner in which new information was conveyed, and the relevance of the module structure to the client needs they had identified. This consultation session was followed by a debriefing session with the study team and technical advisors, who summarised input from peer supporters, added their own considerations, and discussed a roadmap for adaptation and eventual implementation. After the end of the Youth Summit, the research team synthesised and integrated specific feedback into the intervention content and implementation plan, producing a finalised intervention package to prepare for implementation.

### Phase 3 findings

Feedback from the two consultation meetings drove significant changes to the **Boost** component, specifically regarding stigma-related considerations as well as logistical implementation concerns.

#### Reframing terminology.

While content was, overall, described as relevant and acceptable, peer supporters and technical advisors advocated strongly for the framing of the **Boost** modules to be less explicitly linked to mental health, due to stigma and anticipated reluctance of young mothers to participate in such a programme. Instead of using language focused on ‘depression’ and ‘mental health,’ concepts were reframed to encompass ‘stress,’ ‘sadness,’ and ‘support.’

#### Addressing stigma and increasing inclusion.

Related to this concern, it was also suggested that the **Boost** component be delivered to groups of young mothers living with HIV, instead of individually. This request was pragmatic, allowing peer supporters to reach more young mothers in a more structured way, and was also stigma-sensitive. Confidentiality was still a paramount concern, but groups were proposed to begin with confidentiality debriefings to ensure all participants were aware of ground rules. These groups were proposed to take place in private rooms in health facilities or outdoors if such rooms were not available. These changes, while significantly changing the structure of the intervention and its envisioned delivery, reflected significant inputs on the part of implementers and supervisors.

#### Streamlined format.

A new version of the **Boost** component was developed, based on this feedback, simplifying some of the original ideas and content (see Box 2). While the eight sessions remained structured around the same themes as the earlier iteration, the new group-based format loosened recruitment criteria to increase inclusivity, so that new participants could join any group at any time, and not be excluded on the basis of past non-participation (e.g. the intervention did not need to be sequential). All content designed to be individually administered was broadened to guide the peer supporter through a group-based session.

Prompts remained to help peer supporters transition between topics and identify certain negative thought patterns to target, however, these were more geared towards structuring the group interaction. Furthermore, the participant choice that was built into the original intervention concept—enabling individual participants to choose between specific unhealthy thoughts that they related to, to interrogate further—was removed to facilitate smoother group engagement and allow the peer supporter to deliver the intervention with more flexibility and autonomy. Participant packs were retained and distributed to all participants as they joined.

## Discussion

This paper describes the process of adapting and co-developing an evidence-based component, **Boost**, within a broader peer-delivered intervention, ABCD, to improve psychosocial wellbeing among young mothers living with HIV. It adds to a growing literature that seeks to engage young people in developing interventions (Shahmanesh et al., 2021), in this case through helping to formulate a non-clinical, non-specialist support programme that enables under-resourced healthcare systems to provide differentiated services to at-risk populations (Baumel et al., 2018; Bernays et al., 2020; Mavhu et al., 2020; McLeish & Redshaw, 2017). Our approach to co-development was strengthened by two related factors: the process of consultation and co-creation, and integration with existing programming.

First, we drew on the expertise of young mothers living with HIV themselves, as well as the intended implementers, young peer supporters, who were also living with HIV and in some cases were young parents themselves. While incorporating the perspectives of end-users and specifically young people is often cited as critical to intervention success (Oliveras et al., 2018; Simmons et al., 2021), this practice is not consistently adopted (Rufurwadzo et al., 2020). A recent systematic review of psychosocial interventions to improve health engagement of adolescents and young people living with HIV found that just over half of interventions involved adolescents in the planning, design, or implementation of the intervention (Laurenzi et al., 2021a). Rather than being an exercise in expanding participation, this engagement with end-users fundamentally shifted the shape and focus of our intended intervention. Ultimately, these iterations created an intervention component that had strong buy-in among peer supporters and young mothers themselves because it was driven by the needs of the target group along the continuum of project design and adaptation. Just as consultations were central to the development of the original *Thinking Healthy* model, this engagement enabled our adapted **Boost** component to reflect the needs of young mothers living with HIV in our country settings more closely, though further refinements may be needed.

Secondly, we were able to effectively layer this programme onto PATA’s peer support model, taking advantage of an existing infrastructure of well-trained young people, clinic-based mentorship, and in-country organisational relationships (Hatane et al., 2018). Integrating health services across multiple facilities or between facility- and community-based settings has been found to be effective in other under-resourced contexts (Chuah et al., 2017; Phiri et al., 2017). The peer supporter model became the driving force behind the collaborative development of ABCD, and enabled access to a diverse cadre of young people living with HIV, and the young mothers who they supported, alongside their mentors and supervisors. The rich feedback from these stakeholders resulted in the creation of a more acceptable, relevant intervention package, integrated with PATA’s programme, to support psychosocial wellbeing.

These two factors facilitated the development process in particular, ensuring time was allocated for the consultations, content design, and testing necessary to prepare an intervention of this magnitude. In parallel, we used this co-development period to establish plans for training and implementation. As intervention specialists and programme implementers increasingly adopt practices rooted in co-development and adaptation, our experience offers a useful examination of applying implementation science principles in ‘real-world’ programmatic settings—and adapting an evidence-based intervention across lower- and middle-income countries from a general maternal population to adolescent and young mothers living with HIV. It also provides a blueprint that other research teams may adopt when working with community-based organisations, pairing end-user consultations and iterative co-development with a systems-integrated approach to ensure the intervention is contextually relevant and its potential benefits are maximised.

This process faced some limitations, for example, in adhering to a rapid timeline and with limited opportunities for in-depth data collection and consultation at each country site ahead of intervention drafting. As we relied on programmatic data, not all countries and implementation teams were able to contribute data for the analyses and process outlined here. While our main aim was to report on the co-development process and outcomes with an implementation science lens, we acknowledge that the process was not representative from a robust research perspective. However, some of the findings we present, including findings about experiences of stigma and intervention components designed to increase inclusivity, align with existing research from all countries (Bernays et al., 2021; Carbone et al., 2019; Hackett et al., 2019; Mackworth-Young et al., 2017). As this first iteration of the **Boost** component and ABCD was piloted simultaneously across four countries, a longer timeframe could have enabled the research team to match content more closely to country-specific considerations—including local language adaptations, and probing differences in young mothers’ needs across multiple country settings as well as between urban and rural sites. Additional analyses of ABCD implementation data, to be shared in a forthcoming manuscript, can provide greater detail on ways in which country-based technical advisors and peer supporters found ways to adapt and implement their programmes to suit the realities they faced. However, we feel that this process still facilitated multiple levels of engagement and input, resulting in a well-received tool for peer supporters.

As the COVID-19 pandemic continues to shape the environment and circumstances in which interventions can be delivered, especially in low- and middle-income countries (Okumu et al., 2021), task-shifting approaches to service provision beyond healthcare facilities are much-needed. This co-developed psychosocial support component **Boost**, integrated within the ABCD peer supporter package, offers a promising approach to reaching young mothers living with HIV who may require additional mental health support.

## Figures and Tables

**Table 1.** Data sources across each phase.

Data source	Phase 1: Formative research	Phase 2: Content adaptation and development	Phase 3: Consultation, refinement and modification
Consultation session notes	X	X	X
Peer supporter initial feedback	X		
Expert consultations	X		X
Scoping review of literature	X	X	
Questionnaires from technical advisors	X	X	
Notes from informal interviews with young mothers	X	X	
Source intervention manual to be adapted	X	X	
Additional debriefing meeting notes			X

## Box 1. Paediatric-Adolescent Treatment Africa’s peer support model.

Paediatric-Adolescent Treatment Africa (PATA) is a non-governmental organisation that has worked with over 400 health facilities, and implemented and evaluated 10 incubation projects in sub-Saharan African countries. PATA has been a longtime advocate of peer support programming, and has supported multiple networks of peer supporters over the past decade (Mark et al., 2019). PATA’s peer supporters are based at health facilities and communities, and deliver adherence and psychosocial support through monthly peer support groups, and linkage to other services. Peer supporters are recruited based on a defined set of criteria; adolescents and young people ages 18–24 openly living with HIV and successfully adhering to treatment and care are eligible. These individuals are trained to focus on HIV treatment literacy and adherence education, sexual and reproductive health and rights, disclosure, and general psychosocial support to clients. Peer supporters also learn skills related to group facilitation, communication, leadership, advocacy, and record-keeping. They become equipped to support bi-directional referrals between facility-based teams and community-based services, identify urgent cases of treatment failure and psychosocial need, and manage ethical dilemmas. Peer supporters are age-matched to their target client population, typically a few years older but still relatable as near-peers. They typically connect to clients through routine clinic visits, and sometimes work as a team if based at the same facility. Most peer supporters actively engage with approximately 20–40 adolescents at any given time. Ongoing in-service support through mentorships, regular supervision and performance review are also provided to peer supporters by their facility-based supervisors and PATA’s country-based technical advisors; they can similarly access job aides, supportive materials and regular feedback from health workers and clients through PATA’s centralised support (learning platform, events, and technical advisors). PATA’s peer supporters, importantly, are integrated as fundamental members of the facility health teams, helping to support the take-up of adolescent-friendly health services and allowing for meaningful engagement between peer supporters and other health workers.

## Box 2. Refined and finalised Boost session structure.

### Session structure:

- Attendance
- Recap of previous session’s theme and topic
- Introduction of the session’s theme through exploring negative/unhelpful thoughts linked to the theme, and probing related feelings and behaviours as a group
- Identifying positive thoughts to replace this negative thought
- Group discussion and individual selection of helpful/positive thoughts to try
- Group activity to put discussion into practice
- Summary of discussion
